# Frailty in Individuals With Mental Disorders: Longitudinal Analyses of All-Cause Mortality

**DOI:** 10.1192/bjo.2022.223

**Published:** 2022-06-20

**Authors:** Julian Mutz, Alexandru Dregan

**Affiliations:** King's College London, London, United Kingdom

## Abstract

**Aims:**

Frailty is a medical syndrome that is strongly associated with mortality risk, and an emerging global health burden. Mental disorders are associated with reduced life expectancy and elevated levels of frailty. In this study, we examined the mortality risk associated with frailty in individuals with a lifetime history of mental disorders compared to non-psychiatric controls.

**Methods:**

The UK Biobank study recruited >500,000 adults, aged 37–73 years, between 2006–2010. We derived the two most common albeit distinctive measures of frailty, the frailty phenotype and frailty index. Individuals with lifetime depression, bipolar disorder or anxiety disorders were identified from multiple data sources. The primary outcome was all-cause mortality. We have also examined differences in frailty, separately by sex and age.

**Results:**

Analyses included up to 297,380 middle-aged and older adults with a median follow-up of 12.19 (IQR = 1.31) years, yielding 3,516,706 person-years of follow-up. We observed higher levels of frailty in individuals with mental disorders for both frailty measures. For key comparisons, individuals with a mental disorder had greater all-cause mortality hazards than their controls. The highest hazard ratio (3.65, 95% CI 2.40–5.54) was observed among individuals with bipolar disorder and frailty, relative to the non-frail controls.

**Conclusion:**

Our findings highlight elevated levels of frailty across three common mental disorders. The increased mortality risk associated with frailty and mental disorders represents a potentially modifiable target for prevention and treatment to improve life expectancy.

